# Assessment of arterial stiffness variables in patients with rheumatoid arthritis: A mediation analysis

**DOI:** 10.1038/s41598-019-41069-5

**Published:** 2019-03-14

**Authors:** Delia Taverner, Silvia Paredes, Raimon Ferré, Lluís Masana, Antoni Castro, Joan-Carles Vallvé

**Affiliations:** 1 Secció de Reumatologia, Servei de Medicina Interna, Hospital Universitari Sant Joan, Institut Investigació Sanitària Pere Virgili, Reus, Catalonia Spain; 2 Facultat de Medicina, Universitat Rovira i Virgili, CIBER de Diabetes y Enfermedades Metabólicas Asociadas, Institut Investigació Sanitària Pere Virgili, Reus, Catalonia Spain; 3 Facultat de Medicina, Universitat Rovira i Virgili, Servei de Medicina Interna, Hospital Universitari Sant Joan, CIBER de Diabetes y Enfermedades Metabólicas Asociadas, Institut Investigació Sanitària Pere Virgili, Reus, Catalonia Spain

## Abstract

We aimed to study arterial stiffness variables in patients with rheumatoid arthritis (RA), specifically considering their associations with path model mediation analysis. We examined arterial stiffness expressed by the pulse wave velocity (PVW), augmentation index (AIx), distensibility, and clinical and biochemical characteristics in a cohort of 214 RA patients. Variable associations were analysed using multivariate linear regression analysis. We also used path model mediation analysis for PWV variable. Our results indicate that age, systolic blood pressure (SBP), and body mass index (BMI) were significantly associated with PWV, and collectively accounted for 32% of PWV variability. The parallel mediation analysis showed that SBP and BMI accounted for 21% and 7% (a total of 28%) of the total effect of age on PWV, respectively, indicating a partial mediation effect. The associated variables with AIx were age and tender joint count, while those with distensibility were BMI and sex, overall accounting for 16.5% and 4.7% of the variation in AIx and distensibility, respectively. We observed no associations of arterial stiffness with inflammatory variables, disease activity and duration, or cholesterol levels. In conclusion, in our population of RA patients, age is the most important variable that determines the increase in PWV. We have also shown that a significant proportion of the negative effects of age on PWV occurs through increases in SBP and BMI. In our study, lipid and inflammation variables were not associated with an increase in arterial stiffness.

## Introduction

The increase in cardiovascular risk (CV) is currently one of the most important causes of comorbidities in patients with rheumatoid arthritis (RA)^1,2^. The chronic inflammation that acts as an independent risk factor for CV^3^ seems to increase the morbidity and CV mortality of these patients^4,5^.

Increased arterial stiffness is one of the earliest stages of atherosclerosis^6^ and the measurement of pulse wave velocity (PWV) is widely accepted as a precise and non-invasive method to assess arterial stiffness in humans^7^. While the PWV directly measures the arterial elasticity of the great vessels^8^, the augmentation index (AIx) is a more complex parameter that depends on both vascular elasticity and peripheral resistance.

An additional parameter used to measure arterial stiffness is carotid distensibility, which measures the ability of the artery to expand and contract during cardiac pulsation and relaxation^9^. These indices are frequently used as surrogate CVD endpoints in clinical studies since they provide non invasive measures that reflect early effects of several stimuli on the arterial wall^10^.

In recent years, there has been a growing interest in the relationship between PWV, Alx, and RA due to reports that an increase in these markers of arterial stiffness suggests endothelial dysfunction. Some case-control studies reported increased arterial stiffness in RA patients^11–13^. In these studies, associations with variables that are characteristic of the disease such as disease duration^14,15^, inflammatory variables^16–19^, and disease activity^16,20–22^ have been demonstrated, although arterial stiffness has also been correlated with age^19,23,24^ and SBP^23–25^. There are few published studies that have evaluated distensibility as a marker of arterial stiffness in RA^26^, and the results of these studies are similar to those obtained for PWV and AIx. However, these findings were in contrast to those of other publications, which demonstrates the need for additional studies and meta-analyses to provide information about these associations^11,17,27^. Furthermore, others and we have evaluated subclinical atherosclerosis in RA^28–30^ but the relationship of arterial stiffness parameters in individuals with subclinical atherosclerosis has shown differing results^31,32^.

The aim of this study was to describe the association between arterial stiffness as measured by PWV, AIx, and carotid distensibility by specifically considering the relationship between the characteristics of the disease and lipid profiles within a population of RA patients, as well as mediating variables that might affect that relationship. Additionally, we sought to determine if there was a relationship between arterial stiffness, cIMT, and carotid plaque presence in RA patients.

## Materials and Methods

The AR population of the present study has been described in a previous paper^30^. Briefly, the 1987 American College of Rheumatology criteria for RA diagnosis were used to select patients who attended the University Hospital Sant Joan de Reus via external consultation. 214 patients between 20 and 80 years of age were included in the study. The clinical research Ethics Committee of the University Hospital Sant Joan approved the study and we executed the investigation in accordance with our Institution’s guidelines and the Helsinki Declaration.

We informed patients about the research nature of the study and they provided written informed consent prior to participation. Patients were included from September 2011 to November 2014 and on the same day of the visit, fasted blood extraction and carotid ultrasound were carried out.

### Laboratory measurements

Fasting venous blood samples were obtained in EDTA tubes from 214 patients. Analytical variables including hemogram, general biochemistry, glycated haemoglobin, thyrotropin, albumin, and lipid profile [triglycerides (TG), total cholesterol (TC), low-density lipoprotein cholesterol (LDLc), high-density lipoprotein cholesterol (HDLc), and very-low-density cholesterol (VLDLc)] were determined by enzymatic methods. Measurement of apolipoproteins A1 and B were carried out by immunonephelometry and lipoprotein (a) by an enzyme-linked immunosorbent assay. Standard laboratory methods were used to quantify rheumatoid factor (RF), citrullinated anti-cyclic peptide antibodies (ACPA), antinuclear antibodies, and inflammatory markers [erythrocyte sedimentation rate (ESR), C-reactive protein (CRP), and fibrinogen]. RF positive (RF+) was defined by values of RF >20 and positive ACPA (ACPA+) was defined by ACPA values >1. Dyslipemia was defined as having HDLc <50 mg/dL for women or <40 mg/dL for men, or TG >150 mg/dL, or LDLc >100 mg/dL or in treatment with statins or other hypocholesterolemic drugs.

### Clinical evaluation

We obtained information about the classical CV risk factors (smoking, hypertension, diabetes, and hypercholesterolemia), the use of hypolipidaemic, hypoglycaemic, or antiplatelet drugs, and history of CV events. Additionally, we performed measurements of body weight, height, body mass index (BMI), waist circumference (WC), systolic blood pressure (SBP), diastolic blood pressure (DBP), and joint physical examinations of RA. The disease activity score (DAS28) calculated according to the ESR was obtained as a measure of disease activity. Determination of swollen and tender joint counts were also obtained (TJC, SJC respectively). The 0–10 visual analogue scale and the health assessment questionnaire (HAQ) index were used as a measure of pain and disability, respectively.

### Ultrasound evaluation of arterial stiffness and intima-media thickness

Arterial stiffness expressed by the pulse wave velocity, carotid distensibility and augmentation index was measured directly at both common carotid arteries using the ultrasound linear probe (5–12 MHz) as a tonometer and analysed *in vivo* by the Quality Arterial Stiffness (QAS©) radiofrequency software (Esaote SpA, Genova, Italy). Radiofrequency (RF) Signal-based Vascular Ultrasound from Esaote employs RF signal-based technology and includes QAS measurement. The RF signal is a reflected ultrasound signal that is captured by the transducer and converted in an electric signal preserving all the characteristics of the acoustic wave in terms of amplitude and phase. Local arterial stiffness is estimated as systo-diastolic changes in arterial diameter/area over systo-diastolic changes in distending pressure (pulse pressure). Maximum and minimum carotid diameters were acquired using the attained distension curves, and vascular stiffness parameters were calculated after calibration for blood pressure^33,34^. Carotid distensibility was the change in diameter of the carotid artery secondary to intravascular volume expansion caused by the left ventricle systole. The pulse wave velocity was obtained from brachial blood pressure and the accurate measurements of diameter and change in diameter of carotid arteries. Augmentation index was measured by the pulse wave analyses and local pressure. Final values were the median measurements of the right and left carotid arteries^35,36^.

Carotid intima-media thickness (cIMT) was measured with a My Lab 50X-Vision sonographer (Esaote SpA, Genova, Italy) with a linear array ultrasound probe small parts broadband transducer (5–12 MHz). The far wall of the common carotid artery (1 cm proximal to the bifurcation), the bifurcation, and the internal carotid artery of the left and right carotid arteries were identified. We measured cIMT *in vivo* at the predefined points using the QIMT© radiofrequency image processing software (Esaote SpA, Genova, Italy). We defined pathological cIMT as the 75th percentile of cIMT values in the general population banded with respect to age and sex, and plaque as a focal structure encroaching into the arterial lumen by at least 0.5 mm or 50% of the surrounding IMT value, or a thickness >1.5 mm. A single operator obtained and measured the images in order to reduce variability

### Statistical Analysis

**Figure 1:**
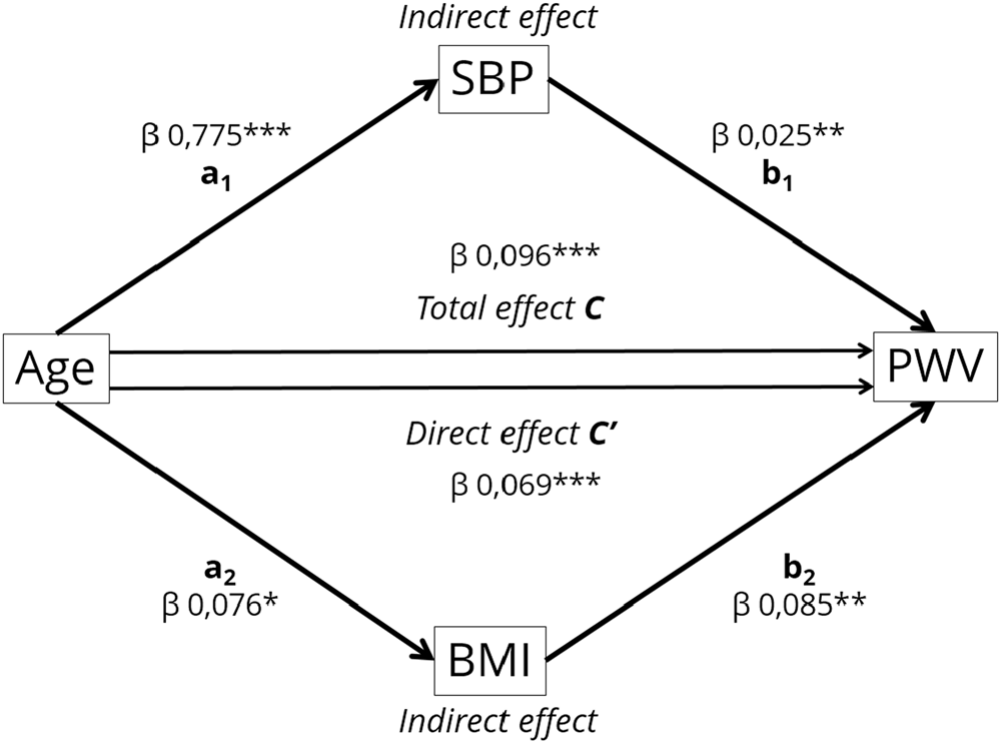
Path model diagram with the results of parallel mediation analysis. Path model showing the effect of age on the PWV as mediated simultaneously by SBP and BMI. Total β effect (path c) represents the effect of age on PWV with no mediators in the model. Direct β effect (path c’) represents the effect of age on PWV when SBP and BMI mediators are included in the model. Indirect effects (path a_1_b_1_ or path a_2_b_2_) represent the effect of age on PWV through SBP or BMI respectively. The figure shows unstandardized β regression coefficients (*p < 0.05, **p < 0.001, ***p < 0.0001).

Statistical software SPSS, version 23 was use to analysed all data. Continuous variables are presented as the mean (standard deviation), and categorical variables are presented as the percentage (number of individuals). Bivariate correlations were estimated using the Pearson correlation coefficient “r”. Multiple linear regression with univariate and multivariate models were used to estimate PWV, AIx, and distensibility variables. We initially selected clinically relevant variables and known confounders for inclusion in the multivariable regression analysis, and the iterative process of variable selection was performed using stepwise logistic regression analysis. Data for AIx was only available for 128 RA patients. The effect of disease activity degree was assessed by interaction analyses. Interaction terms of the four levels of DAS28 (remission, low, moderate, and high disease activity) with the variables to be tested were added in the linear regression models. The interaction terms were considered significant when significantly improved the variability of the dependent variable explained by the model. Mediation analysis was used to further understand the association between age and PWV through the use of the Hayes macro-application “PROCESS” for SPSS^37^. Specifically, we used a parallel mediation model (model number 4 in Hayes macro) to examine total, direct, and indirect effects of age on PWV (Fig. 1). The path coefficients are the beta (β) coefficients of the multivariable regression models and represent the magnitude and direction of associations between variables included in the model. The total β effect (path c) represents the effect of age on PWV when no other mediators are included in the model, while the direct β effect (path c’) represents the effect of age on PWV when mediators are included. Finally, indirect effects (path a_1_b_1_ and path a_2_b_2_) represent the effect of age on PWV through SBP or BMI, respectively. If the indirect effects path is statistically significant, it can be concluded that mediation has occurred. The significance of indirect effects are tested by bootstrapped 95% confidence intervals (CI). Process macro produces bootstrap estimates and bias-corrected 95% confidence intervals (CIs) for the indirect effect, and a 95% CI that does not cross zero indicates a statistically significant indirect. The effect size for mediation analysis was measured with percent mediation (P_M_) interpreted as the percent of the total effect (path c) accounted for by the indirect effects (path a_1_b_1_ or path a_2_b_2_). For the other tests, a p-value of <0.05 was considered statistically significant.

### Ethical approval and informed consent

The study was approved by the clinical research Ethics Committee of the University Hospital Sant Joan and it was carried out in accordance with our Institution’s guidelines and the Helsinki Declaration. Patients were informed of the research nature of the study and provided written informed consent prior to participation

## Results

We included 214 patients with RA in the study. Table 1 shows the general characteristics of the cohort included. Mean (SD) age and disease duration were 58 (12) and 9.4 (9.1), respectively. Female patients represented 64.5% of the cohort; 60% of patients were hypertensive, 11.7% were diabetic, 41% were dyslipidaemic, and 26% were smokers. The percentage of patients in remission or with low, moderate or high disease activity were 27, 19, 44, and 10, respectively.

**Table 1:** General characteristics of RA patients.

	RA (n = 214)
**Characteristics of the groups**	
Sex-female (%, n)	64.5 (138)
Age (years)	58 (12)
Body mass index (kg/m^2^)	27.8 (5.9)
Waist circumference (cm)	93 (15)
Systolic blood pressure (mmHg)	137 (21)
Diastolic blood pressure (mmHg)	81 (12)
LDL cholesterol (mg/dL)	119 (31)
HDL cholesterol (mg/dL)	66 (19)
Triglycerides (mg/dL)	105 (55)
Glucose (mg/dL)	95 (23)
Current smoker (%, n)	26.2 (56)
Hypertension (%, n)	60.3 (129)
Diabetes mellitus (%, n)	11.7 (25)
Dyslipidemia (%, n)	41.1 (88)
**Disease features**	
Disease duration (years)	9.4 (9.1)
DAS28	3.5 (1.3)
Disease Remission (%,n)	27.1 (58)
Low disease activity (%, n)	18.7 (41)
Moderate disease activity (%, n)	44.4 (95)
High disease activity (%, n)	9.8 (21)
HAQ	0.45 (0.52)
Rheumatoid factor + (%, n)	72.4 (155)
ACPA + (%, n)	81.3 (174)
ESR (mm/h)	37 (26)
CRP (mg/dL)	0.72 (0.83)
Fibrinogen (mg/dL)	443 (97)
**Treatments (%, n)**	
DMARDs	75.2 (161)
Biological agent	20.1 (43)
NSAIDs	57 (122)
Corticosteroids	50.9 (109)

HAQ; health assessment questionnaire index, ACPA; citrullinated anti-cyclic peptide antibodies, ESR; erythrocyte sedimentation rate, CRP; C-reactive protein, DAS28; disease activity score, DMARDs; disease-modifying antirheumatic drugs, NSAIDs; non-steroidal anti-inflammatory drugs.

Disease-modifying antirheumatic drugs were administered to 75% of the patients which included 20% of patients receiving biological drugs, 57% receiving non-steroidal anti-inflammatory drugs, and/or 51% receiving corticosteroids.

Crude analysis of the ultrasonographic variables showed that ESR, BMI, age, and DBP were significantly correlated with PWV and distensibility, while SBP was only correlated with PWV. Moreover, PWV was significantly correlated with disease duration, CRP, LDLc, TG, glucose, and HbA1c (Table 2). Finally, AIx was significantly correlated with disease duration, HDLc, age, and SBP (Table 2). Nevertheless, many of these bivariate correlations no longer remained significant after adjustment for confounder variables in multivariate models (shown below). Thus, stepwise multivariable linear regression analysis showed that the variables significantly associated with PWV were age (β = 0.069; p = 0.0001), SBP (β = 0.022; p = 0.0003), and BMI (β = 0.078; p = 0.0002), collectively explaining 32% of PWV variability (R^2^ = 0.32; p < 0.0001). No other variable was associated with PWV. Then we tested the heterogeneity in disease activity degree of our population by interaction analyses between DAS28 and age, SBP, and BMI. When we added the different interaction terms in the model, we observed no significant effects of those terms on the variability of PWV explained by the model (Supplemental Table 1).

**Table 2:** Correlations of arterial stiffness variables with clinical and analytical variables.

	Pulse wave velocity			Distensibility			Augmentation index		
	r	β	p	r	β	p	r	β	p
Disease duration (years)	0.118	0.001	0.048	−0.01	0.001	0.447	0.146	0.073	0.050
DAS28	0.010	0.001	0.442	−0.036	0.001	0.305	−0.008	−0.028	0.463
HAQ	0.031	0.006	0.33	−0.087	0.006	0.108	−0.029	−0.254	0.374
ESR (mm/h)	0.212	0.001	0.001	−0.139	0.001	0.024	0.049	0.009	0.293
CRP (mg/dL)	0.118	0.016	0.047	−0.065	0.016	0.177	−0.142	−0.843	0.056
Fibrinogen (mg/dL)	0.071	0.0008	0.159	−0.015	0.0001	0.415	0.005	0.0001	0.477
HDLc (mg/dL)	0.006	0.0001	0.465	0.018	0.0001	0.401	0.240	0.059	0.003
LDLc (mg/dL)	0.156	0.001	0.013	−0.043	0.001	0.272	0.079	0.012	0.188
Triglycerides (mg/dL)	0.132	0.0001	0.031	−0.009	0.0001	0.448	−0.008	−0.001	0.463
Age	0.514	0.005	0.0001	−0.140	0.005	0.023	0.371	0.152	0.0001
BMI (kg/m2)	0.298	0.006	0.0001	−0.181	0.006	0.005	0.059	0.055	0.254
SBP (mmHg)	0.447	0.002	0.0001	−0.038	0.002	0.295	0.185	0.040	0.019
DBP (mmHg)	0.192	0.002	0.003	−0.140	0.002	0.023	0.059	0.021	0.255
Glucose (mg/dL)	0.199	0.001	0.002	−0.060	0.001	0.199	−0.001	0.0001	0.494
HbA1c (%)	0.177	0.024	0.006	0.001	0.024	0.492	0.013	0.065	0.443

r; spearman coefficient; p; p value; β; univariate linear regression estimates.

DAS28; disease activity score, HAQ; health assessment questionnaire index, ESR; erythrocyte sedimentation rate, CRP; C-reactive protein, HDLc; High-density lipoprotein cholesterol, LDLc; Low-density lipoprotein cholesterol, BMI; Body mass index, SBP; systolic blood pressure. Data for AIx was only available for 128 AR patients.

To further explore the relationships between age, SBP, and BMI with PWV, we performed a parallel mediation analysis (Fig. 1; Table 3). The model showed that the total effect of age (c pathway) on the dependent variable (PWV) was mediated by SBP (a_1_b_1_ indirect pathway) and by BMI (a_2_b_2_ indirect pathway). Thus, age was positively associated with SBP (path a_1_) and with BMI (path a_2_). Additionally, SBP was also positively associated with PWV (path b_1_), while BMI was positively associated with PWV (path b_2_). Bootstrapping analysis revealed a significant indirect effect of age on PWV through SBP (a_1_b_1_ = 0.0203 (95% CI; 0.0082, 0.0338)) and through BMI (a_2_b_2_ = 0.0065 (95% CI; 0.0006, 0.0149)) as the 95% CI did not cross zero. The total indirect effect ((a_1_b_1_) + (a_2_b_2_)) was 0.0268, (95% CI; 0.0125, 0.0425) with a stronger contribution from SBP than from BMI. Combined, both mediators accounted for approximately 28% of the total effect of age (P_M_ = 0.279), with SBP accounting for 21% of the total effect (P_M_ = 0.212) and BMI accounting for 6.7% of the total effect (P_M_ = 0.067). The model also showed a significant direct effect (path c’) of age on PWV indicating that the cumulative mediating effect of SBP and BMI was only partial (partial mediation), as the total effect of age on PWV was reduced (β coefficient changed from 0.096 to 0.069) but remained significant.

**Table 3:** Parallel mediator model summary.

	Pulse Wave velocity (m/s)				
	β	SE	P value	95% CI	
				Lower	Upper
**Age**					
Total effect (path c)	0.096	0.0123	0.0001		
Direct effect (path c’)	0.069	0.013	0.0001		
Age to SBP (path a_1_)	0.775	0.11	0.0001		
Age to BMI (path a_2_)	0.076	0.032	0.0182		
Total indirect effect	0.0268			0.0125	0.0425
**SBP**					
Direct effect (path b_1_)	0.025	0.0075	0.001		
Indirect effect (path a_1_b_1_)	0.0203			0.0082	0.0338
**BMI**					
Direct effect (path b_2_)	0.085	0.026	0.001		
Indirect effect (path a_2_b_2_)	0.0065			0.0006	0.0149

Data are obtained from PROCESS macro-application (37). Total β effect (path c) represents the effect of age on PWV with no mediators in the model. Direct β effect (path c’) represents the effect of age on PWV when SBP and BMI mediators are included in the model. Indirect effects (path a_1_b_1_ or path a_2_b_2_) represent the effect of age on PWV through SBP or BMI respectively.

SBP: systolic blood pressure; BMI: body mass index; SE: standard error; β: linear regression estimates.

Stepwise multivariable linear regression analysis showed that BMI (β = −3.31; p = 0.022) and sex (β for men = 36.27; p = 0.024) were significantly associated with distensibility, accounting for 5.6% of overall distensibility variability (R^2^ = 0.056; p < 0.003). Furthermore, stepwise multivariable linear regression analysis showed that age (β = 0.14; p = 0.0001) and TJC (β = −0.33; p = 0.045) were significantly associated with AIx, accounting for 16.5% of overall AIx variability (R^2^ = 0.165; p < 0.0001). No interaction effect of DAS28 on distensibility neither AIx was observed (Supplemental Table 1). We then checked for the association between PWV, distensibility, and AIx with cIMT and carotid plaque presence. We showed through multivariable regression analysis that there was no association of these variables with either cIMT or plaque presence (Supplemental Table 2).

## Discussion

In this study, we evaluated PWV, AIx, and carotid distensibility as markers for arterial stiffness in a cohort of 214 patients with RA. Our results identified variables that were significantly associated with each marker: age, SBP, and BMI accounted for 32% of the overall variability in PWV; age and TJC accounted for 16.5% of the overall variability in AIx; and BMI and sex accounted for 5.6% of the overall variability in distensibility. In addition, interaction analyses shown that these associations did not change as a function of DAS28.

The clinical relevance of an increase in PWV and AIx can be understood more clearly when one considers that the risk of a major CV event increases by approximately 14% for every 1 m/s increase in PWV. Similarly, the risk of a major CV event increases by 31.8% for every 10% increase in AIx^38^.

Furthermore, through a mediation analysis, we decomposed the total effect of age on PWV into a direct effect of age and into an indirect effect through other variables previously associated with PWV. We showed that 28% of the total age effect on PWV is driven by SBP (21%) and BMI (7%), while the remaining 72% of the total effect can be attributed exclusively to age. The cumulative mediating effect of these variables is only partial, as the total effect of age on PWV is reduced but remained significant. It is plausible that the negative effect of age on PWV is mediated by increased SBP and BMI as these variables have been shown to independently affect PWV.

As seen in this study, age, SBP, and BMI have been previously identified as independent predictors of arterial stiffness in RA patients^21,23,24,39^. However, the effect of age on PWV, mediated by SBP and BMI, has not been previously described. On the other hand, distensibility is not as well-studied as a marker of arterial stiffness in RA patients^25,26^, and the association between BMI and sex observed in our study has not been previously published.

Treatment with NSAIDs or corticoids to which our patients are subjected, may affect the associations found in our study. In fact, it has been previously described that NSAIDs and corticosteroids are associated with arterial stiffness^40–42^. In any case, in our study, when “NSAIDs treatment” or “corticoids treatment” variables were introduced into the models, there was no significant association with PWV, AIx, or distensibility. Even so, the fact that corticosteroids promote endothelial dysfunction and increase the production of reactive oxygen species^42^ does not rule out corticosteroids as possible negative effectors of arterial stiffness. Studies designed with this objective would be necessary to better evaluate the effect of these treatments on arterial stiffness.

Additionally, we observed no significant association between inflammatory variables (CRP, ESR, and fibrinogen), disease activity or disease duration and arterial stiffness (PWV AIx, and distensibility). This is in agreement with previous studies^19,23,31,43–45^ but in contrast with others^11,14,16,17,20–22^. These discrepancies might be attributed to the different levels of inflammatory biomarkers profiled and to the relatively short mean disease duration of nine years within our cohort.

High cholesterol levels have been associated with reduced arterial elasticity^22,39^, but in our study as in others^19^, no association was found with lipid profiles. Therefore, static measurements of classical lipid values might not be sufficient to generate clinically useful information until more advanced measures are utilized (i.e., sizes and concentrations of lipid subfractions, compositional changes, and functionality).

In a previous study with this population^30^, we showed that age was associated with cIMT and plaque presence. In the same way, age has been the variable more associated with arterial stiffness in the present study showing the importance of this variable in the development of atherosclerotic process. On the other hand, in our previous study, we also observed an age-gender interaction effect on cIMT, which has not been observed in the present study on arterial stiffness. Furthermore, in the previous report, DAS28 was associated with an increase in cIMT in women but in the present study, we did not find any association of arterial stiffness with RA disease severity. These differences show that although cIMT and PWV are markers of early arterial wall alteration, the remodeling process of the arterial wall is still incompletely understood in RA patients. Furthermore, PWV and AIx have been independently associated with cIMT and plaque presence in RA patients^31^, an observation that has not been detected in the present study. Moreover, other studies failed to find this association, and the question of whether arterial stiffness augments cardiovascular risk by increasing atherosclerosis remains unclear^6,32^.

Our study has some limitations: its cross-sectional nature do not allow us to draw any conclusion about the causal relation between PWV, distensibility, and AIx and atherosclerosis. Furthermore, the selection of our RA patients are regional focus, so the results might be too population-specific and difficult to generalize across other populations. Thus, larger series with measures of atherosclerosis and with follow-up would be needed to confirm a clinical relevance of an increase in PWV and AIx regarding CV risk in our RA patients.

In conclusion, the results of this study demonstrate that in patients with RA, age is the most important variable for determining the increase in PWV. We also found that a significant proportion of the negative effect of age on PWV occurs through an increase in SBP and BMI. Finally, we found no association between lipid or inflammation variables with an increase in arterial stiffness.

## Supplementary information

Supplemental information

## Data Availability

The datasets generated during the analysis of the current study are available from the corresponding author on request.
