# Supplementary material for: Assessment of arterial stiffness variables in patients with rheumatoid arthritis: A mediation analysis
Source: Sci Rep. 2019 Mar 14;9:4543. doi: 10.1038/s41598-019-41069-5 (PMC6418197; doi:10.1038/s41598-019-41069-5)
Supplement: Supplementary file 1 — Supplemental information [file 41598_2019_41069_MOESM1_ESM.pdf]

Assessment of arterial stiffness variables in patients with rheumatoid arthritis: A mediation analysis

Delia Taverner, Silvia Paredes, Raimon Ferré, Lluís Masana, Antoni Castro, Joan-Carles Vallvé.

Supplemental table 1: Association of interaction terms with PWV, Distensibility, and Augmentation index.

	PWV (m/s)				
Interaction term	β	SE	P value	95%CI	
Age*DAS28	0.00031	0.002	0.89	-0.004	0.179
BMI*DAS28	0.0011	0.005	0.82	-0.009	0.011
SBP*DAS28	0.0002	0.001	0.83	-0.002	0.002
	Dist (m/s)				
Interaction term	β	SE	P value	95%CI	
Age*DAS28	0.00031	0.002	0.89	-0.004	0.179
BMI*DAS28	0.0011	0.005	0.82	-0.009	0.011
	AIx				
Interaction term	β	SE	P value	95%CI	
Age*DAS28	0.014	0.008	0.068	-0.001	0.03
TJC*DAS28	-0.227	0.198	0.254	-0.62	0.16

Adjusted β linear regression estimates of the effect of interaction terms on PWV, Dist, and AIx.
PWV; pulse wave velocity, Dist; distensibility, Aix; augmentation index, β; linear regression estimates, SE; standard error, CI; confidence interval, DAS28; disease activity score, BMI; Body mass index, SBP; systolic blood pressure, TJC; tender joint counts. Data for AIx was only available for 128 AR patients.

Supplemental Table 2: Association of arterial stiffness variables with cIMT and plaque presence.

		**PWV**	**AIx**	**Distensibility**
**cIMT**				
	**β**	2.48	-2.35	0.039
	**SE**	3.49	1.17	0.065
	**95% CI**	-4.4; 9.38	-5.86;1.16	-0.09;0.17
	**R^2 (%)**	9	1.3	0.1
	**P value**	0.47	0.18	0.55
**Plaque presence**				
	**OR**	1.003	1.05	1.002
	**SE**	0.09	0.048	0.002
	**95% CI**	0.84;1.2	0.96;1.16	0.99;1.005
	**R^2 (%)**	8.1	6.7	0.8
	**P value**	0.97	0.28	0.14

Adjusted β linear regression estimates and OR of the effect of PWV, AIx and distensibility on cIMT and plaque presence. Models are adjusted for age, gender, exercise, disease duration, body mass index, systolic and diastolic blood pressure. SE=standard error. OR=odds ratio.
